# Supplementary material for: Exploring the Multifunctional Roles of Odontoglossum Ringspot Virus P126 in Facilitating Cymbidium Mosaic Virus Cell-to-Cell Movement during Mixed Infection
Source: Viruses. 2021 Aug 6;13(8):1552. doi: 10.3390/v13081552 (PMC8402721; doi:10.3390/v13081552)
Supplement: Supplementary file 1 [file viruses-13-01552-s001.zip › viruses-1296607-supplementary.pdf]

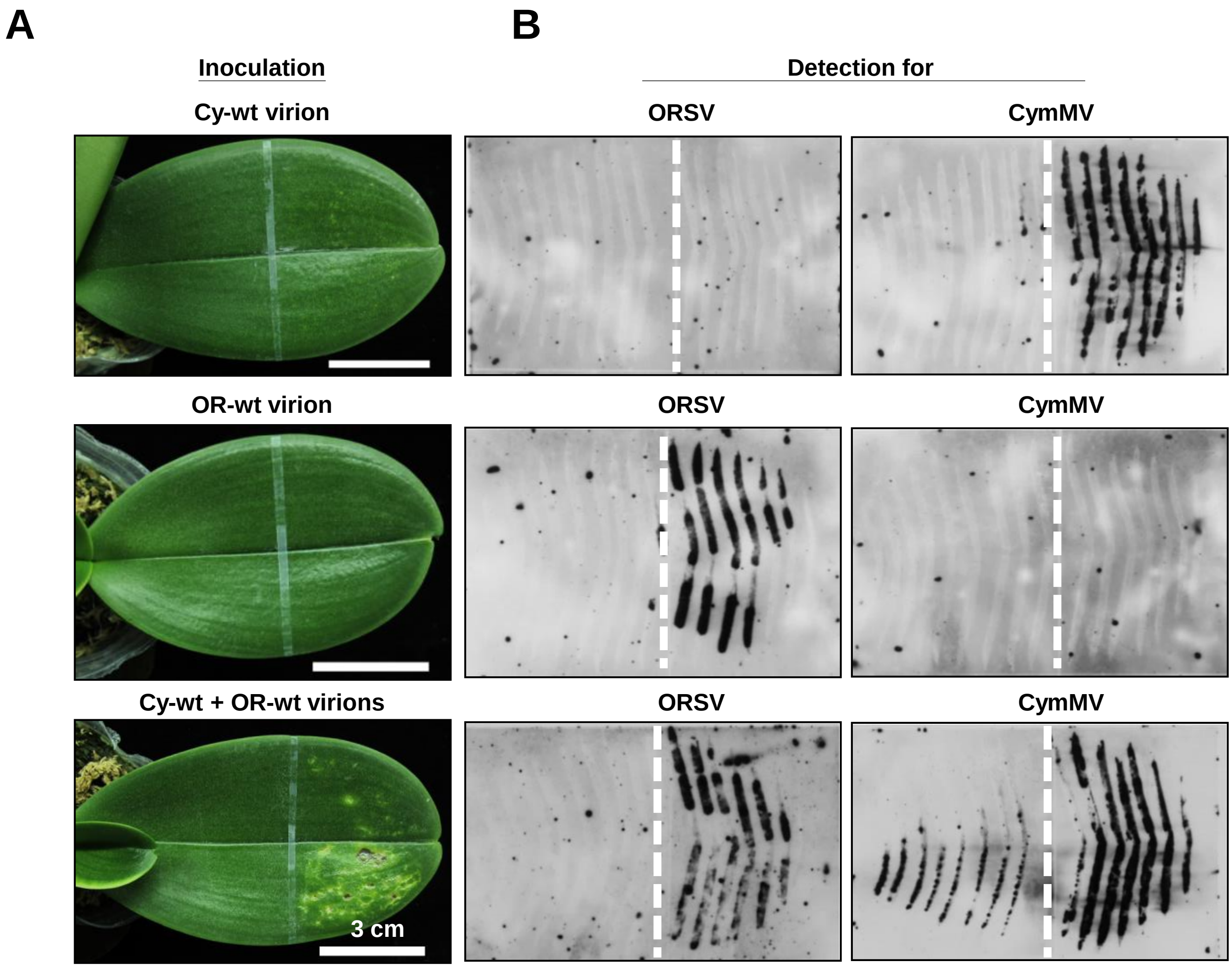


**Figure S1**. Infectivity of wild type isolates of ORSV and CymMV in *Phalaenopsis* orchids. (A) The virions of wild type CymMV (Cy-wt) and ORSV (OR-wt) were used to assay infectivity and their synergistic effects upon co-infection. Four-months after deflasking *Phalaenopsis* plantlets, they were inoculated individually with 1 μg virions/leaf of CymMV or ORSV or co-inoculated with CymMV and ORSV (0.5 μg/leaf each). Symptoms were photographed at 10 DPI. (B) Tissue blots, prints of sliced IL in (A), were exposed to DIG-labeled probes against the CP and 3′ UTR regions of the CymMV and ORSV genomes, respectively.

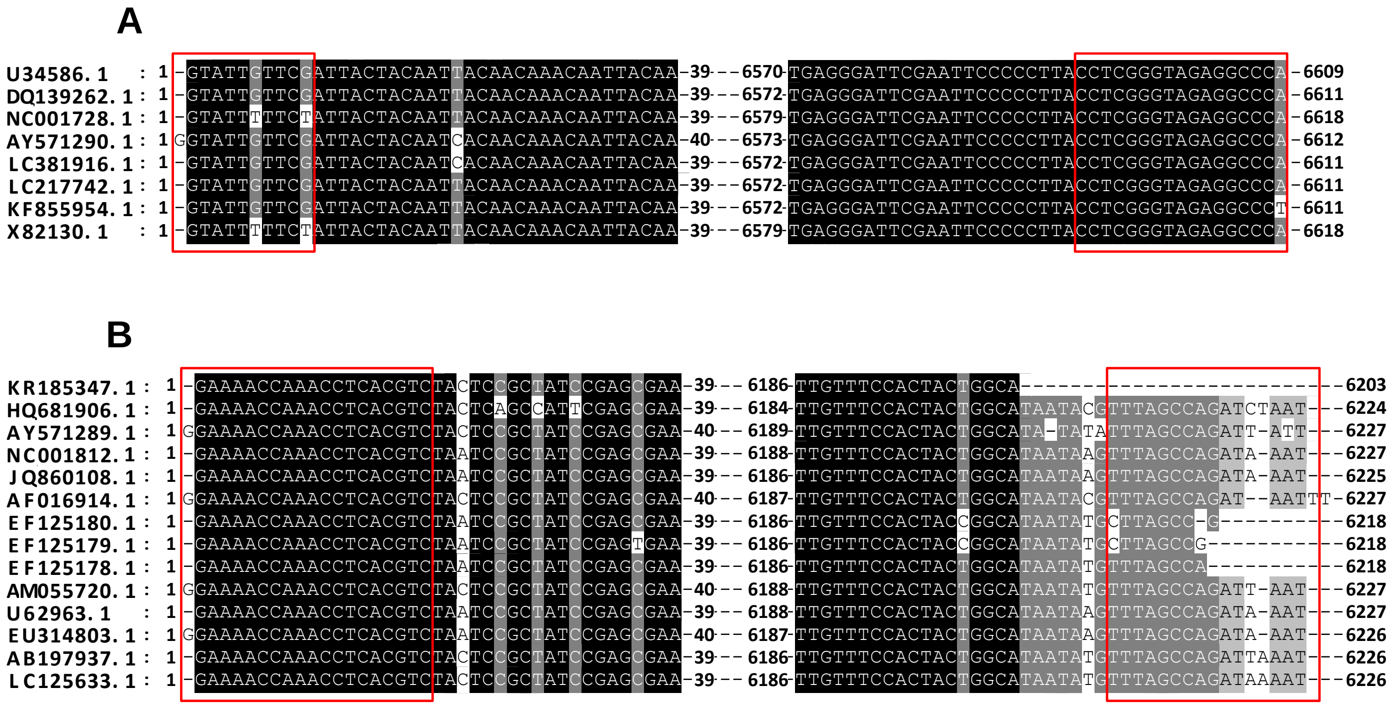

**Figure S2.** Sequence alignments of ORSV and CymMV UTRs. ORSV and CymMV genome sequences obtained from the NCBI database. Forty nucleotides of the ORSV (A) or CymMV (B) 5′ and 3′-ends have been aligned. The conserved sequences (red boxes) were used for primer design to amplify the full-length ORSV and CymMV genomes, respectively. Accession numbers for each full-length viral genome are listed in the left panel.

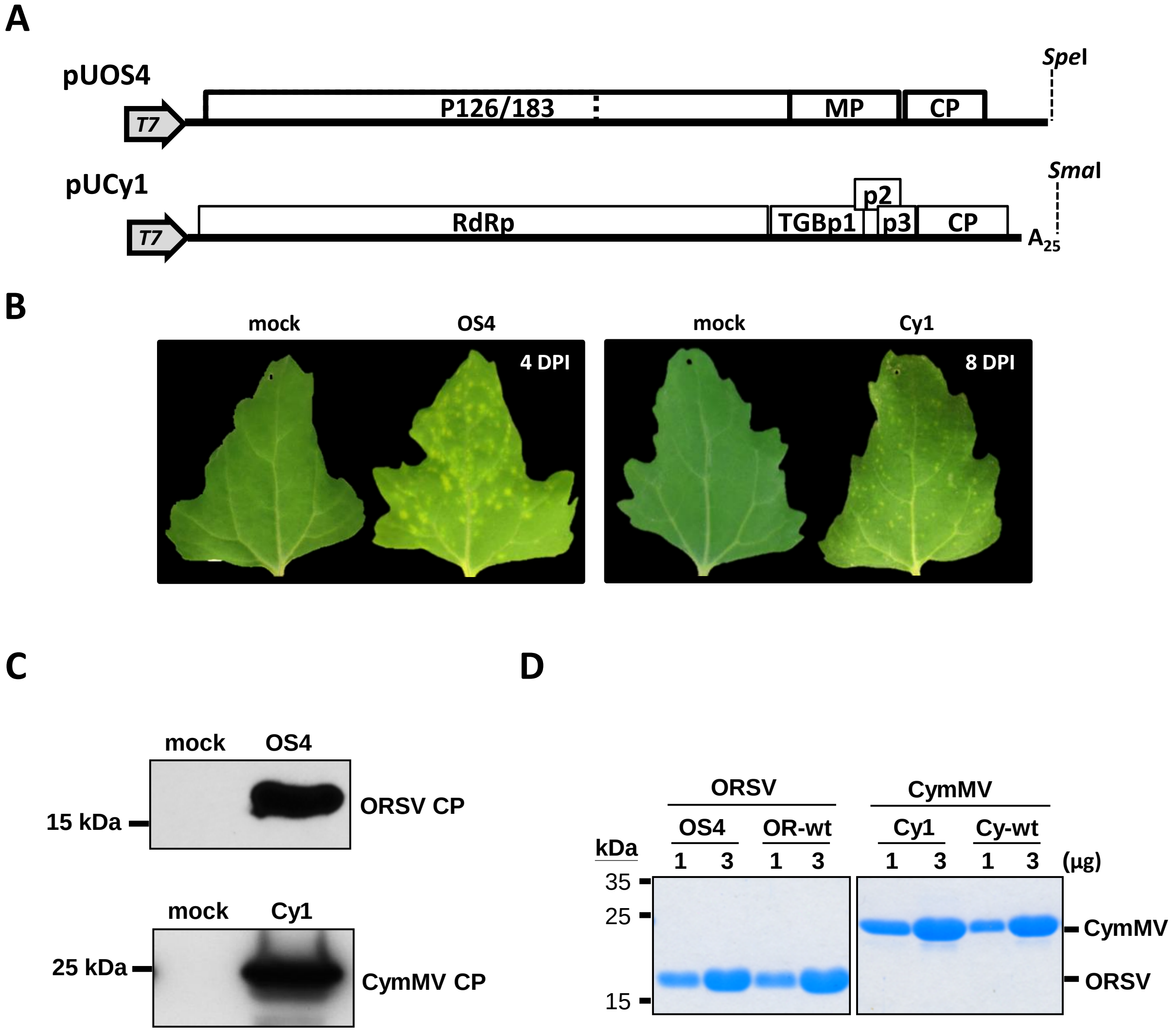


**Figure S3**. Genome maps of viral cDNA clones, pUOS4 and pUCy1 and their infectivity in *C. quinoa*. (A) Genome organization of full-length CymMV and ORSV cDNAs, pUCy1 and pUOS4. (B) Localized lesions elicited on *C. quinoa* by RNA transcripts at 4 DPI for pUOS4 and 8 DPI for pUCy1. (C) Immunodetection of the OS4 and Cy1 capsid proteins (CPs) from leaf extracts of inoculated plants at 7 and 14 DPI, respectively, using antibodies against the CPs of CymMV or ORSV, respectively. (D) SDS-PAGE of purified ORSV and CymMV virions derived from infectious cDNA clones (OS4 and Cy1) and viruses isolated from diseased orchids (OR-wt and Cy-wt). One and three micrograms of OS4 and OR-wt or Cy1 and Cy-wt virions were loaded in parallel as a control. Protein sizes are indicated at left and corresponding viral CPs are labeled at right.

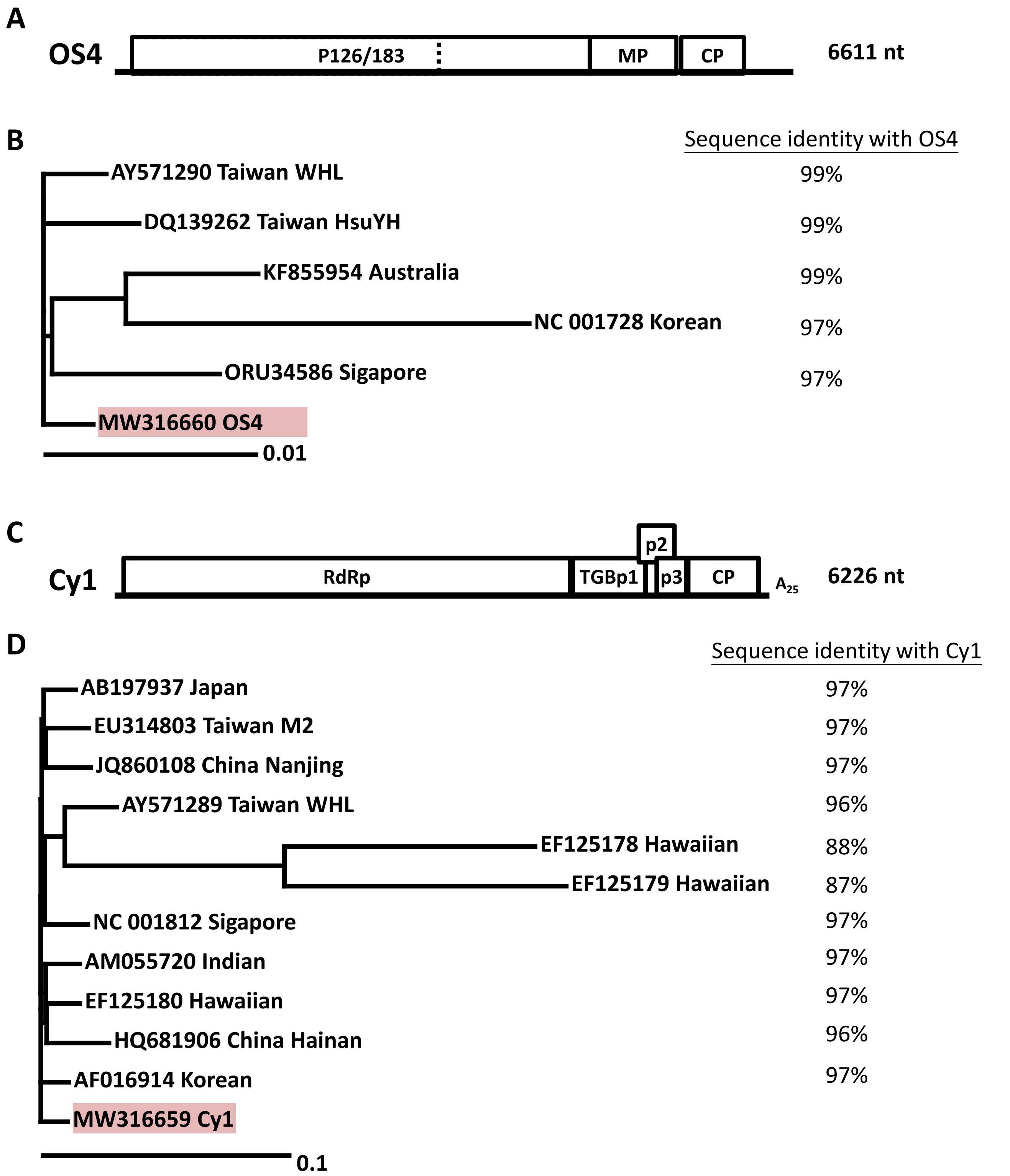


**Figure S4**. Sequence analysis of ORSV and CymMV cDNA clones. (A) Schematic representation of OS4 genome organization. Locations of three ORFs plus one readthrough ORF are shown and arranged in scale. (B) Phylogenetic analysis of OS4 and ORSV isolates from the NCBI database. (C) Schematic representation of Cy1 genome organization. Locations of five ORFs are arranged in scale. (D) Phylogenetic analysis of Cy1 and CymMV isolates from the NCBI database. The accession numbers of individual ORSV or CymMV isolates are listed and their sequence identities to the OS4 or Cy1 genomes are also shown. All alignments were analyzed using the AlignX program in Vector NTI Advance 10.3.0 (Invitrogen Co., USA).

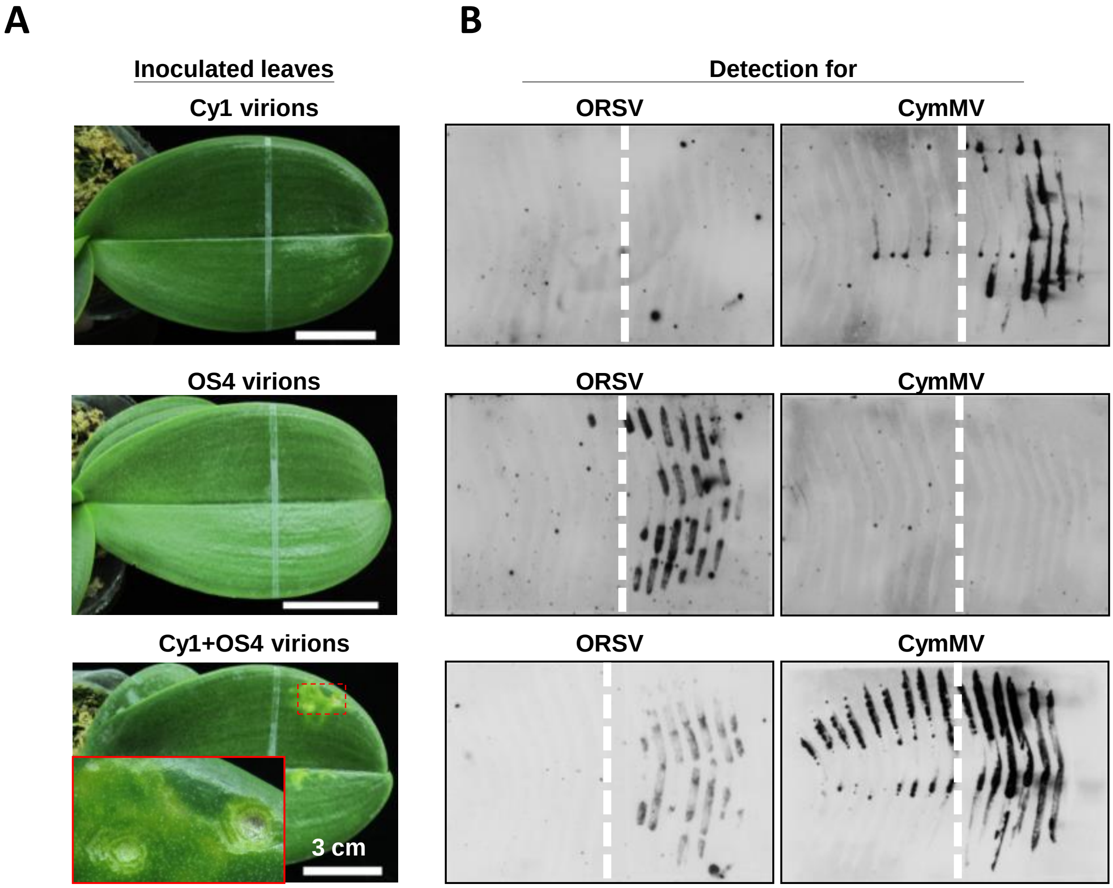


**Figure S5.** Cy1 and OS4 infectivity and synergism in *Phalaenopsis* orchids. (A) Cy1 and OS4 virion infectivity and their synergistic effects upon co-infection in *Phalaenopsis* orchids were assayed as described in Fig. S1. Symptoms were photographed at 10 DPI. Red box at bottom is a manganite image of the necrotic lesions. (B) Tissue blotting to detect CymMV and ORSV RNAs.

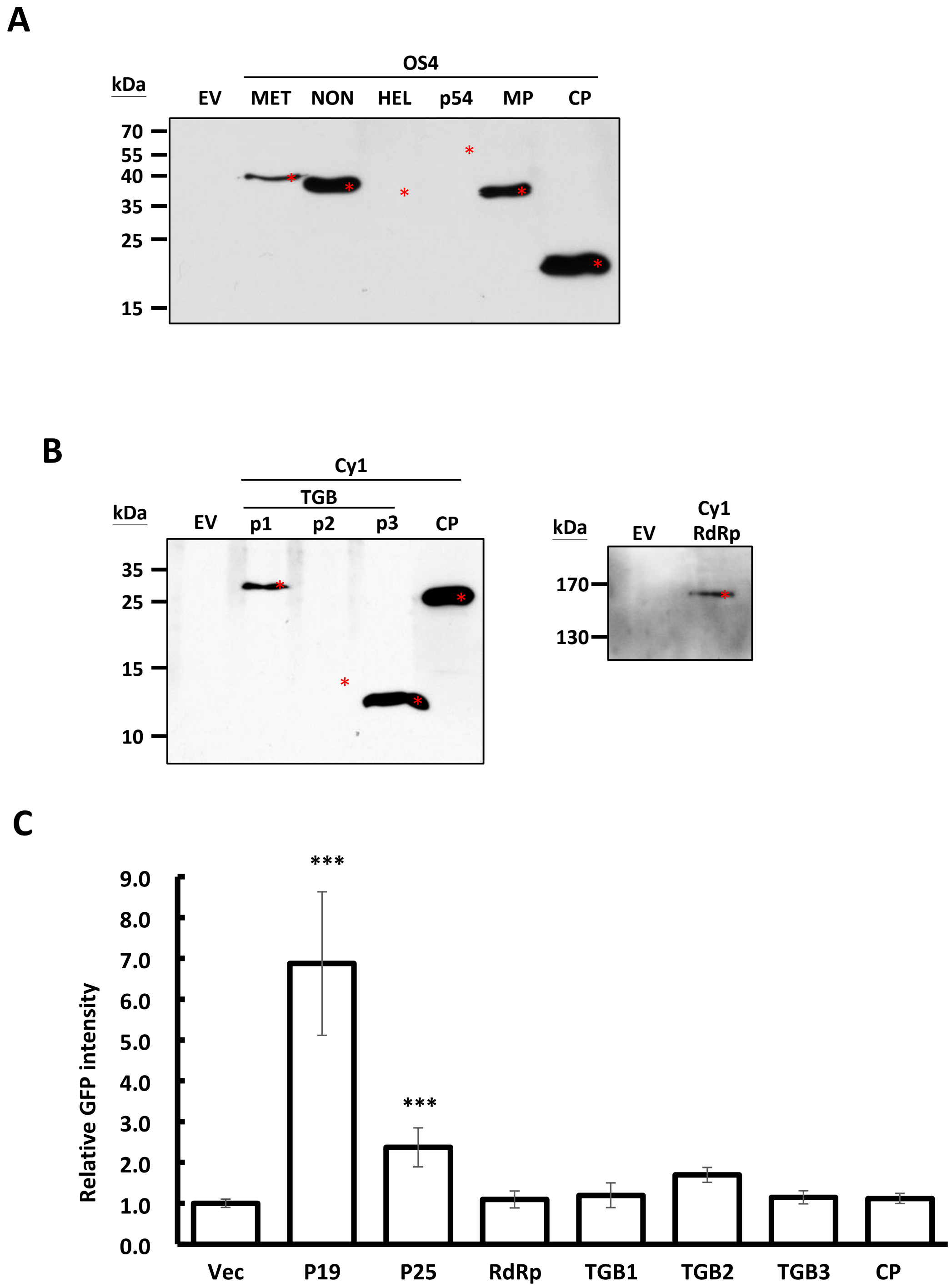


**Figure S6.** Accumulation of overexpressed viral proteins and VSR activity assay for CymMV proteins. *Agrobacterium* carrying pBIN61 vector (EV), or pBIN61-viral proteins were infiltrated into 3-week-old *N. benthamiana* leaves, respectively. Accumulation of (A) ORSV and (B) CymMV proteins were assessed by western blot using rabbit anti-HA antibody at 2 DPA. EV: vector control. (C) GFP signal was detected and quantified using IVIS Lumina III LT *in vivo* Imaging System at 4 DPA. Relative GFP intensity represents the mean of three independent experiments and was normalized to vector control. The significance was calculated by Student's $t$ test and marked when $p < 0.001$ (***).

**A**

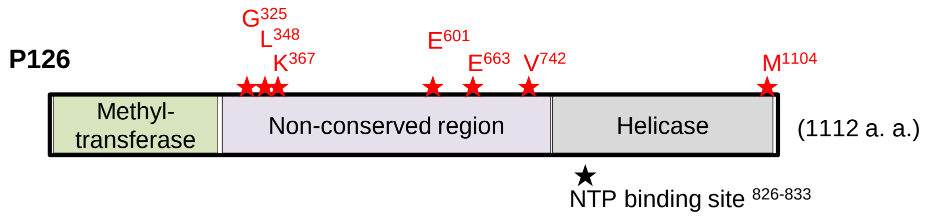


**B**

| Protein | VSR activity | Protein | VSR activity |
|---|---|---|---|
| P126 | + | P126R (M$^{1104}$R) | – |
| <u>P126-like mutants</u> | | <u>P126R-like mutants</u> | |
| E$^{601}$A | + | G$^{325}$A, M$^{1104}$R | – |
| E$^{663}$A | + | L$^{348}$A, M$^{1104}$R | – |
| V$^{742}$A | + | K$^{367}$A, M$^{1104}$R | – |
| P126A (M$^{1104}$A) | + | E$^{601}$A, M$^{1104}$R | – |
| P126S (M$^{1104}$S) | + | E$^{663}$A, M$^{1104}$R | – |
| | | V$^{742}$A, M$^{1104}$R | – |

**C**

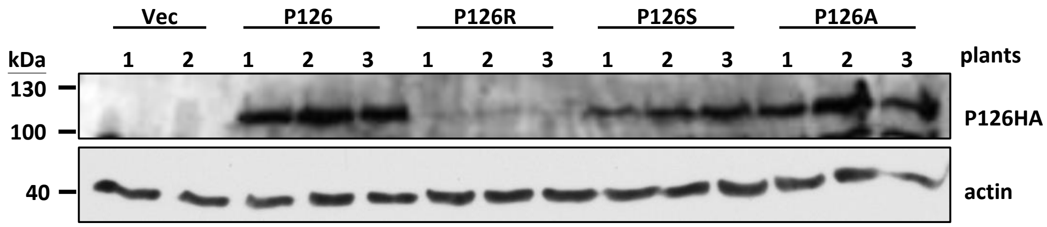


**Figure S7.** VSR assays of ORSV P126 and its mutants. (A) Schematics representing targeted amino acids for mutation and their positions in P126. Functional domains and mutation sites (red stars) of ORSV P126 are labeled. (B) Single and double mutants of P126 generated by mutagenesis categorized into two types, P126-like or P126R-like, and their respective VSR activities. +: displays VSR activity; -: lacks VSR activity. (C) Protein accumulation of P126 and P126 mutants. *Agrobacterium* carrying pBIN61 vector (Vec), pBIN61-P126 (P126) or its derivate mutants (P126R, P126S and P126A) was infiltrated into 3-week-old *N. benthamiana* leaves, respectively. Accumulation of P126 and P126 mutant proteins at 2 DPA was assessed by western blot using anti-HA antibody. Actin acted as a loading control. Vec: vector control. Each lane represents the results of an individual plant.

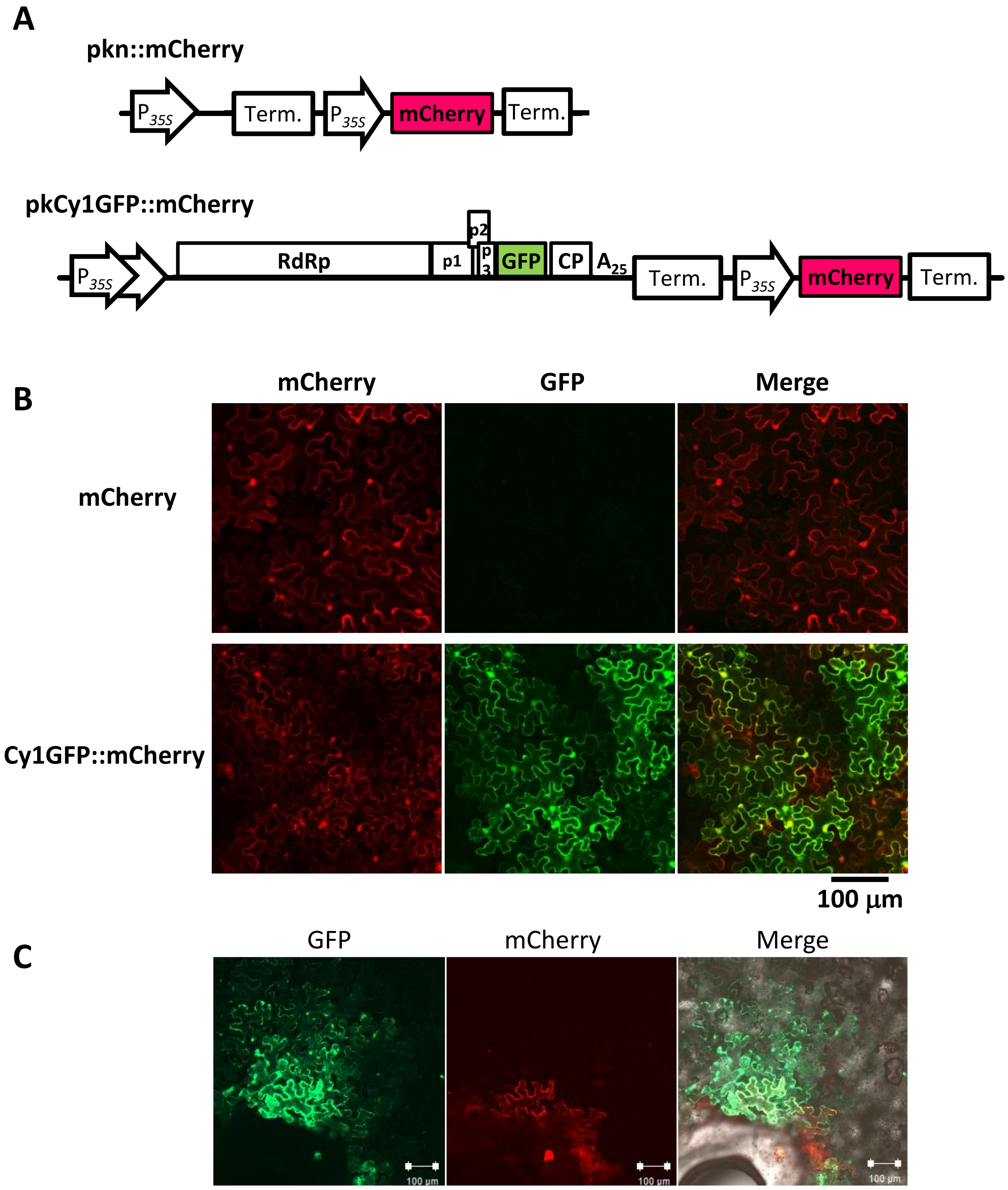


**Figure S8.** Expression cassettes used to determine the primary infection foci and cell-to-cell movement of Cy1GFP. (A) Schematics of pkn::mCherry and pkCy1GFP::mCherry. (B, C) Representative images of infection of whole *N. benthamiana* leaves (B) or by pinpricking (C) using *Agrobacterium* carrying pkn::mCherry or pkCy1GFP::mCherry. The *Agrobacterium* infection site (mCherry) and Cy1GFP (GFP) spread were observed by confocal microscopy at 4 DPA.

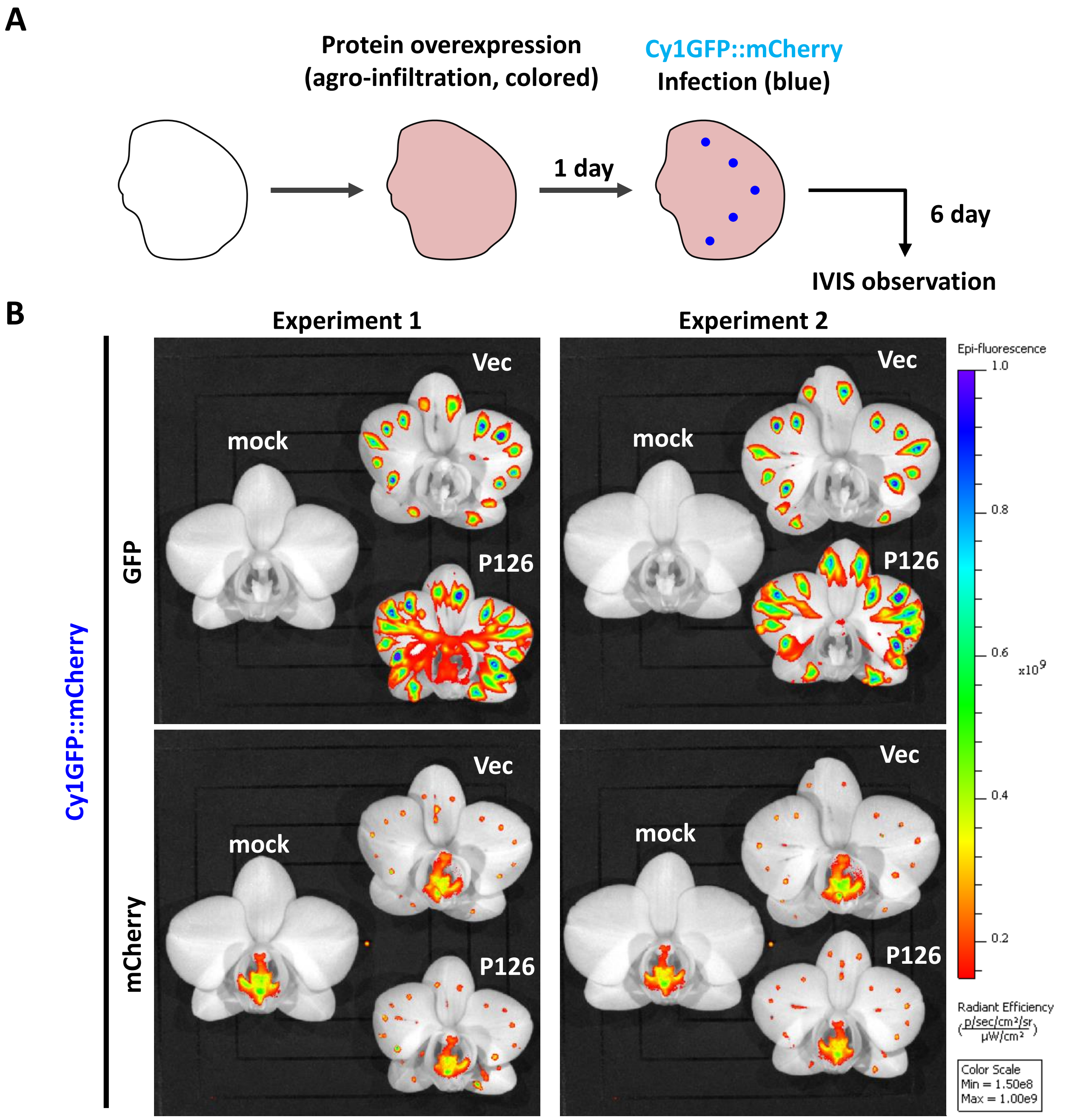


**Figure S9.** CymMV cell-to-cell movement upon overexpressing P126 in *Phalaenopsis* flowers. (A) Flowchart for protein expression and CymMV inoculation in *Phalaenopsis* flowers. After 1 day post-agroinfiltration, the *Agrobacterium* ooze carrying pkCy1GFP::mCherry was collected on a toothpick and pinpricked onto the infiltrated flowers. (B) Representative images of *Agrobacterium* primary infection sites (mCherry) and Cy1GFP spreading infected cells (GFP) using the IVIS Lumina III LT in vivo Imaging System at 6 DPA.
